# Does Type 1 Diabetes Modify Sexuality and Mood of Women and Men?

**DOI:** 10.3390/ijerph15050958

**Published:** 2018-05-11

**Authors:** Ewelina Bak, Czeslaw Marcisz, Sylwia Krzeminska, Dorota Dobrzyn-Matusiak, Agnieszka Foltyn, Agnieszka Drosdzol-Cop

**Affiliations:** 1 Department of Nursing, Faculty of Health Sciences, University of Bielsko-Biala, 43-309 Bielsko-Biala, Poland; 2 Department of Gerontology and Geriatric Nursing, School of Health Sciences, Medical University of Silesia, 40-752 Katowice, Poland; klinwewtychy@poczta.onet.pl; 3 Department of Clinical Nursing Faculty of Health Sciences, Medical University, 50-367 Wroclaw, Poland; s.krzeminska@wp.pl; 4 Department of Nursing Propaedeutics, School of Health Sciences, Medical University of Silesia, 40-752 Katowice, Poland; dorotadobrzyn@op.pl; 5 Department of Social and Humanistic Studies, Chair of Philosophy and Bioethics, School of Health Sciences, Medical University of Silesia, 40-752 Katowice, Poland; agnieszka_foltyn@wp.pl; 6 Chair of Woman’s Health, School of Health Sciences in Katowice, Medical University of Silesia, 40-752 Katowice, Poland; cor111@poczta.onet.pl

**Keywords:** sexual disorders, type 1 diabetes, depression, illness acceptance

## Abstract

Background: Sexual disorders occurring in women and men with type 1 diabetes have not been sufficiently investigated and described until now. This study attempts to evaluate sexuality in women and men. Methods: Altogether, the study comprised 115 patients with type 1 diabetes and 105 healthy people constituting the control group. All the studied persons underwent survey studies determining sexuality using the Female Sexual Function Index (FSFI-19) in women and the International Index of Erectile Function (IIEF-15) in men, and the occurrence of depression using the Beck Depression Inventory. The acceptance of illness among patients with diabetes was examined using the Acceptance of Illness Scale questionnaire. Results: In 35% of the examined women with diabetes, the study demonstrated sexual dysfunction as determined by total FSFI. The point values of all the investigated FSFI domains were significantly lower in women with diabetes than in healthy ones (*p* < 0.001). Erectile dysfunction occurred in 50% of the studied men with diabetes and in 23% of the control group of men (*p* = 0.0017). Conclusions: Type 1 diabetes leads to sexual disorders which occur in 1/3 of women and in 1/2 of men. Sexual disorders in patients with diabetes more frequently occur in men, persons with coexisting complications of diabetes, and in those with a concentration of glycated hemoglobin higher than 6.5%.

## 1. Introduction

Despite the fact that the etiopathomechanism of diabetes and its complications has been fairly thoroughly explored and that in, the light of obtained study results, it was shown that diabetes three times more frequently affects sexual function among men, the complexity of the sexual cycle among women and, moreover, erectile dysfunction still remains a silent complication due to the insufficient number of studies [1,2,3,4]. It should be added that in the case of men with diabetes, erectile dysfunction occurs as much as 10–15 years earlier in comparison with healthy persons [5,6]. The etiopathogenesis mechanism of erectile dysfunction in patients with diabetes is complex and it is most frequently the result of the accumulation of many factors [7]. Sexual disorders among women and men may be modified, i.e., by factors such as: age, psychical condition, and partner relationships [8,9]. Despite over 40 years of studies related to the occurrence of sexual disorders in women with diabetes, these data still remain incomplete and inconclusive [10]. Sexual functioning is an important, though sometimes ignored, component of human well-being [11]. The studies describing the frequency of occurrence of female sexual disorders showed different data: from 14 to 85% [12,13,14,15,16,17,18]. A risk factor for the development of sexual disorders in women which is especially underlined is the psychological aspect [19]. And according to the current knowledge regarding the causes of sexual disorders, in men the source of such disorders should be sought in the somatic and psychological condition [19].

The study results presented in the literature which include the assessment of sexual disorders in women and in men with type 1 diabetes, taking into consideration the psychological and the somatic factors, are negligible and differentiated depending on the applied study methods. This observation became the basis for carrying out the present study, which aims to analyze the influence of the psychical condition, concentration of glycated hemoglobin (HbA1c), duration of diabetes, body mass index, age, and subjective acceptance of illness on sexual disorders in women and men with type 1 diabetes.

## 2. Materials and Methods

### 2.1. The Studied Subjects

The cohort study comprised a population of 115 patients with type 1 diabetes, including 57 women aged 18–49 (31.5 ± 8.9) (Group I) and 58 men aged 18–45 (28.2 ± 7.1) (Group II); and also 105 healthy persons constituting the controls including 43 women aged 18–39 (27.7 ± 6.8) (Group CI) and 62 men aged 18–40 (29.5 ± 6.9) (Group CII). The selection of patients for studies was random. They were selected out of consecutive patients with type 1 diabetes of a diabetic clinic. The patients from Group I were selected out of 111 randomly chosen women with type 1 diabetes. The patients from Group II were selected out of 115 randomly chosen men with type 1 diabetes. The inclusion criteria to the groups of studied patients were the occurrence of type 1 diabetes lasting for at least a year, prior sexual initiation and the occurrence of menstrual periods in women. The persons excluded from the study were patients in whom any inflammation occurred during the last 3 months; patients taking immunosuppressive drugs, glucocorticoids, anti-inflammatory drugs, psychotropic drugs as well as patients with diagnosed cancer, endocrine gland diseases, psychological diseases, alcoholism, and patients who did not provide consent for performing the study. The treatment of diabetes generally consisted of adhering to a diet and taking insulin; some patients received additional oral antidiabetic medications (biguanides, sulphonylureas). All the studied subjects were patients of the Diabetic Units and the Diabetic Out-patient Clinics in the Silesia Province of Poland. The research was carried out in the period from March 2016 until January 2017. The research was carried out with the consent of the Bioethics Committee (No. of consent: 2016/02/11/1).

### 2.2. Methods

The values of the carbohydrate parameters (fasting glycemia, and the concentration of HbA1c) and lipid parameters (the concentration of low density lipoprotein LDL-, high density lipoprotein HDL-, total cholesterol, and triglycerides) were determined in all the studied persons. Systolic blood pressure (SBP) and diastolic blood pressure (DBP) were measured and the mean arterial pressure (MAP = DBP + (SBP − DBP)/3) was calculated. 

Next, questionnaire studies were carried out using the following questionnaires: the patients’ demographic and clinical data survey, Female Sexual Function Index (FSFI), International Index of Erectile Function (IIEF), Beck Depression Inventory (BDI), and Acceptance of Illness Scale (AIS).

#### 2.2.1. The Patients’ Demographic and Clinical Data Survey

The questionnaire was accompanied by brief information about the studied person including parameters such as age, sex, place of residence, education, marital status, professional activity, body mass, height, used stimulants (e.g., coffee, cigarettes, alcohol), comorbidities, the duration of diabetes, the occurrence of diabetes complications (diabetic foot, neuropathy, nephropathy, retinopathy), and taken drugs. In case of the studied women, reproductive history was also assessed.

Before commencing the study, every person was informed about its purpose. The questionnaire was filled in personally and anonymously by the patients during the physician’s visit. The time needed for filling in the survey was 15–20 min.

#### 2.2.2. Female Sexual Function Index (FSFI)

The FSFI questionnaire developed by Rosen et al. [20] consists of 19 questions allowing for the multidimensional assessment of female sexual functions in relation to the period of the last 4 weeks. The index has been standardized and adjusted (in many language versions, including Polish) to differentiating sexual dysfunctions in women aged 18–70 in accordance with the current classifications and recommendations of scientific associations. The questionnaire possesses documented credibility, sensitivity, reliability, and internal consistency, as well as stability and repeatability of results in recognizing disorders of sexual desire, sexual arousal, orgasm, and dyspareunia. The questions presented in the questionnaire have been grouped into six domains: sexual desire, sexual arousal, lubrication, orgasm, sexual satisfaction, and dyspareunia. The final results are obtained separately for each of the subscales by summing up the elementary points which are part of each of the domains and taking into consideration the designated coefficient; the results are also obtained globally (global assessment).

For the evaluation of the particular domains, the point score which may be obtained ranges from 0 to 6.0 for sexual arousal, lubrication, orgasm, and dyspareunia; from 1.2 to 6.0 for the sexual desire function; and from 0.8 to 6.0 for sexual satisfaction. A result below 65% of the maximum score possible to be obtained in each of the domains was considered a sexual disorder of the given function; results below 3.9 points were treated as a sexual dysfunction within a given domain. In the global assessment, the possible score ranges between 2 and 36 points. Sexual dysfunctions are recognized when the total score is equal to or lower than 26.55 [21].

The Cronbach α value of FSFI for our results was estimated at the level of 0.974 for type 1 diabetes patients and 0.959 for controls, showing high reliability.

#### 2.2.3. International Index of Erectile Function (IIEF)

The International Index of Erectile Function (IIEF) is a research tool for the investigation of male sexual functions. IIEF is a multidimensional instrument for 5-grade self-assessment of all the domains of male sexual functions in relation to the period of the last 4 weeks. This index has received standardization for differentiating sexual dysfunctions in men (aged 19–82) in accordance with the international consensus, and it is officially available in 32 language versions, including Polish. It is characterized by high credibility, reliability, sensitivity, and repeatability in the detection of changes, confirmed in over 50 clinical trials. The application of the IIEF in the original or the shortened version (IIEF-5) is a recommended standard in the recognition and assessment of the erectile dysfunction severity grade. The IIEF questionnaire includes 15 items grouped into 5 collective domains (subscales) describing: I—erection (6 questions); II—orgasm (2 questions); III—sexual desire (2 questions); IV—sexual intercourse satisfaction (3 questions); and V—overall sexual satisfaction (2 questions). 

An additional analysis of the subscale referring to erection allows for differentiating four states of severity of erection disorders: lack of erection disorders (26–30 points); and mild (17–25 points), moderate (11–16 points), and severe (6–10 points) erection disorders. A value equal to or lower than 25 points (cut-off point) is considered as the occurrence of clinically significant erectile disorders [22].

The Cronbach α value of IIEF for our results was estimated at the level of 0.930 for type 1 diabetes patients and 0.880 for controls, showing high reliability.

#### 2.2.4. Beck Depression Inventory (BDI)

The Beck Depression Inventory (BDI) is a 21-point screening tool used for assessing the degree of intensity of mood disorder symptoms (depression). The scale consists of 21 questions evaluated from 0 to 3 points. The results obtained in the BDI vary in the range 0–63. A score below 10 points is considered normal. Mild depression is suggested by a score within the range 10–15, moderate depression by 16–23, and severe depression above 24. BDI is a questionnaire standardized and validated for Polish conditions which has been numerously applied in studies assessing mood disorders; it possesses a high α Cronbach reliability coefficient—0.92–0.93 [23]. The Cronbach α value of BDI for our results was estimated at the level of 0.916 for women and 0.857 for men, showing high reliability.

#### 2.2.5. Acceptance of Illness Scale (AIS)

The Acceptance of Illness Scale (AIS) is a questionnaire prepared by Felton et al. and adapted by Juczynski [24]. It includes 8 statements; for each of them the examined patient determines his or her current condition on a five-point scale (from 1 to 5). The total score of all the points is the general measure of the degree of illness acceptance and its scope fits within the range from 8 to 40 points. A low result indicates lack of acceptance, adaptation to the illness, and a strong feeling of discomfort. A high result, in turn, indicates acceptance of the illness state, which manifests itself as a lack of negative emotions related to the illness. 

The Cronbach α value of AIS for our results was estimated at the level of 0.923 for women and 0.908 for men, showing high reliability.

### 2.3. Statistical Analysis

The statistical calculations were performed using Statistica (version 10.0, StatSoft Polska, Cracow, Poland). The *t*-Student test was applied in the analysis of quantitative variables; when none of the variables met the assumptions of the parametric model (normality of distribution, homogeneity of variance), the Mann–Whitney *U* test was used. The tests used for the elaboration of qualitative variables were the Chi-square test, the Yates-corrected Chi-square test, and Fisher’s exact test. Correlation between the FSFI and IIEF (scores) as well as BDI and AIS scales were calculated (Spearman rank correlation test). Additionally, the Mann–Whitney *U* test was used for checking whether the differences between the distributions of data were statistically significant. On the basis of the logistic regression model, it was determined which of the analyzed factors may have a significant influence on the occurrence of sexual functioning disorders and of depressive states in the studied persons. Moreover, the Receiver Operating Characteristic curve (ROC) was designated and the DeLong nonparametric method was used for calculating the Area Under the Curve (AUC). The *p* < 0.05 value was accepted as statistical significance level.

## 3. Results

### 3.1. General Characteristics of the Study Groups

The sociodemographic, clinical, and biochemical characteristics of the studied patients are presented in Table 1.

Both studied groups (Group I and II) were comparable with regard to age with the respective control groups CI and CII, whereas they differed significantly in terms of the body mass index, waist-to-hip ratio, systolic and diastolic blood pressure, fasting glucose, total cholesterol, LDL-cholesterol, and triglyceride levels as compared with healthy controls (*p* < 0.05; Table 1). 

In the reproductive history of the studied women with diabetes, it was demonstrated that the medians of the initiation age (17 years old), menarche (13 years old), the duration of the cycle (29 days) and of menstrual bleeding (5 days), and the number of pregnancies (1) and childbirths (1) were comparable to those found among the women from the control group. Regular menstruation was reported by 74% of women with diabetes and 81% of women without diabetes. The median of the age of sexual initiation in the studied men was the age of 17 in every group with or without diabetes.

### 3.2. Assessment of Sexual Function and Depressive Symptoms in Women

Women with type 1 diabetes had significantly lower scores in all six domains (sexual desire, sexual arousal, lubrication, orgasm, sexual satisfaction, and dyspareunia) of the FSFI, compared to the healthy controls (*p* < 0.01; Table 2). Twenty (35.1%) women with type 1 diabetes and only 2 (4.7%) control women had a total FSFI score ≤26.55, and the total FSFI score was lower in the case of the presence of type 1 diabetes. The mean BDI score was significantly higher in women with type 1 diabetes than in healthy women (*p* = 0.0001). Depressive symptoms (considered together) were experienced by more women with type 1 diabetes (35.1%) than healthy subjects (7%) (Table 2).

### 3.3. Assessment of Sexual Function and Depressive Symptoms in Men

Men with type 1 diabetes had significantly lower scores in four domains (erection, desire, sexual satisfaction, overall satisfaction) of the IIEF compared to the healthy controls (*p* < 0.05; Table 3). The one remaining domain (orgasm) of the IIEF was not statistically significant among the two groups. Twenty-nine (50%) men with type 1 diabetes and 14 (22.6%) controls were diagnosed with erectile dysfunction. Men with type 1 diabetes obtained statistically significantly lower scores in the erection domain. The mean BDI score was significantly higher in men with type 1 diabetes than in healthy men (*p* < 0.001). Depressive symptoms were experienced by more men with type 1 diabetes—22.4% than by healthy subjects—4.8% (*p* < 0.01).

### 3.4. Acceptance of Illness Scale (AIS)

The average value of the AIS in women was 32.5 ± 6.9 points and in men was 33.8 ± 5.9 points. The obtained results are comparable (*p* = 0.4723) and prove the patients’ high level of acceptance of the illness (the AIS scale score range = 8–40 points).

### 3.5. Female Sexual Function Index and International Index of Erectile Function—Correlations in Diabetic Patients

In both women and men with diabetic complications, the mean total FSFI and IIEF scores were significantly lower than in patients without diabetic complications (*p* < 0.001; Table 4).

Type 1 diabetic women with sexual dysfunction had statistically significantly higher levels of HbA1c compared to women without sexual dysfunction (*p* < 0.0001). Among men, no statistically significant differences were found (Table 5).

In diabetic women, the mean total FSFI score and its six domains inversely correlated with the total BDI score (total FSFI: women with diabetes type 1, *r* = −0.65, *p* < 0.0001; healthy subjects, *r* = −0.23, *p* = 0.1306). Similarly, in diabetic men, five IIEF domains inversely correlated with the total BDI score (erection: men with type 1 diabetes, *r* = −0.66, *p* < 0.0001; healthy subjects, *r* = −0.28, *p* = 0.0258) (Table 6).

In both diabetic women and men, FSFI and IIEF scores positively correlated with the total AIS score (total FSFI: *r* = 0.68, *p* < 0.0001; erection: *r* = 0.59, *p* < 0.0001) (Table 6).

The logistic regression analysis of clinically significant FSFI and IIEF values facilitated the marking of the odds ratio (OR) for the occurrence of female and male sexual dysfunction depending on the presence of independent variables: sex, age, BMI, duration of diabetes, HbA1c, complications, and mean arterial pressure (Table 7). The occurrence of sexual disorders significantly depends on the sex (*p* = 0.0096), diabetes complications (*p* = 0.0021), and the concentration of glycated hemoglobin (*p* = 0.0444). The likelihood of diagnosing female and male sexual dysfunction was higher for the male sex (OR = 4.23), HbA1c higher than 6.5% (OR = 1.81), and diabetes complications (OR = 7.91) (Table 7). The likelihood of the occurrence of this sexual dysfunction was over four times higher among men than among women, nearly eight times higher among persons with diabetes complications, and also increased along with the increase of HbA1c concentration. Sexual disorders in patients with diabetes more frequently occurred in men, persons with coexisting complications of diabetes, and when the concentration of HBA1c was higher than 6.5%.

The performed statistical analysis allowed the conclusion that the created logistic regression model proved to be statistically significant; this was confirmed by the result of the likelihood ratio test (χ^2^ = 42.31; *p* < 0.0001). In this model, Nagelkerke’s R^2^ coefficient was 0.42, and the result of the Hosmer–Lemeshow test (*p* = 0.3953) confirmed sufficient goodness of fit.

**Figure 1:**
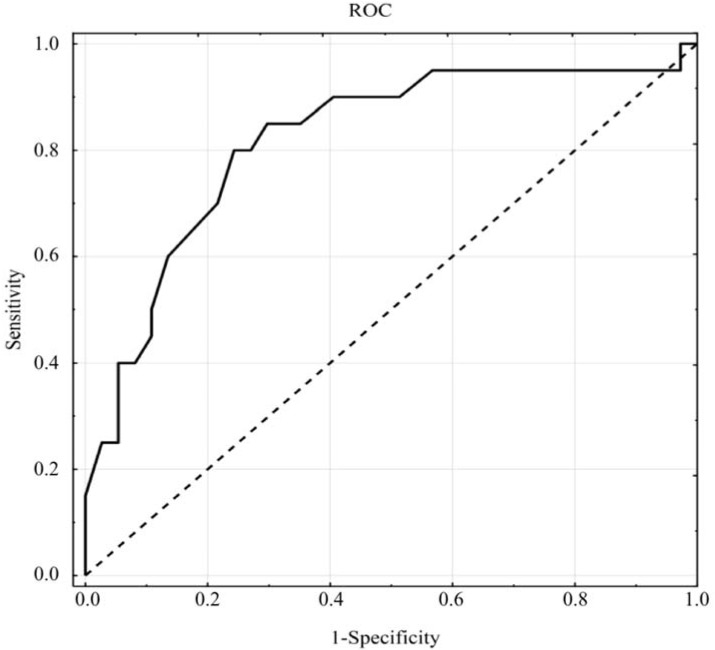
Receiver operating characteristic (ROC) curves of HbA1c for the prediction of female sexual dysfunction.

Although the mean value of HbA1c was low, the HbA1c cutoff was estimated. The analysis of the ROC (Figure 1) showed that HbA1c was an accurate parameter for identifying vulnerable diabetic females (AUC = 0.819 ± 0.062; *p* < 0.0001). Taking HbA1c values lower than 6.5% as a cut-off, HbA1c is able to distinguish sexual dysfunction in diabetic females with a sensitivity of 80% and a specificity of 76%.

## 4. Discussion

Until now, sexuality studies of patients with type 1 diabetes have not identified the risk factors determining the frequent occurrence of sexual disorders in this illness. It has been demonstrated that among men with type 1 diabetes, sexuality was associated mainly with psychological and physical factors, whilst in women, the psychological factor was dominant [19]. The present work aimed to present the frequency of occurrence of sexual disorders in the population of women and men with type 1 diabetes and to determine the potential risk factors for these disorders. The application of commonly applied questionnaires determining sexuality, i.e., FSFI in women and IIEF in men, allowed for demonstrating the occurrence of sexual disorders accompanying type 1 diabetes in connection with the psychological condition (low mood, depression) and with self-acceptance of the illness. In accordance with the total FSFI values obtained by us, sexual disorders occurred in 35% of the population of menstruating women with type 1 diabetes we assessed. A similar percentage (33.8%) of occurrence of sexual disorders in women with type 1 diabetes was demonstrated by Tagliabue et al. [25]. However, the percentage of sexual disorders occurring in studied women with type 1 diabetes observed by Nowosielski et al. [14] was lower (26.5%) and that by Mazilli et al. [18] was higher (51%) than that observed by the authors of the present study. In our previous paper we demonstrated that sexual disorders occurred in as many as 68% of women with type 2 diabetes [17]; however, it needs to be noted that the average age of those women was 20 years higher than that of the women with type 1 diabetes studied by us.

In the present research, the assessment of particular sexual functions in the group of women with type 1 diabetes showed significant differences in all the domains and in the total score when compared to healthy women. In accordance with the data present in the literature, the most frequent sexual dysfunctions occurring in the group of women were, respectively, disorders of sexual desire, sexual arousal, and orgasm [9,26,27]. In our research, it was also demonstrated that among the assessed domains of the sexual functioning of women with diabetes, the most frequent disorder was the weakening of sexual desire and, to a lesser degree—sexual arousal. Other authors did not observe changes in the scope of sexual desire among women with diabetes [10,28,29]. The weakening of sexual arousal in women with diabetes demonstrated in the present paper was also described by Muniyappa et al. [30] and in the compilation of many papers constituting a meta-analysis [31]. Basson et al. [32] demonstrated a lack of a significant influence of diabetes on sexual arousal in the studied women in comparison to the controls. The probable mechanism of the occurrence of sexual arousal disorders in women with diabetes may be associated with the insufficient vascular flow within the pelvis minoris as well as with autonomic and peripheral neuropathy [31]. Orgasm-related disorders observed by us occurred significantly more frequently in the group of women with type 1 diabetes, which proved to be compliant with the study results obtained by Jensen [33]. Doruk et al. [13] in turn observed four times more frequent occurrence of orgasm disorders among studied women with diabetes than we did in our own research. 

In our studies, disorders related to pain (dyspareunia) occurred in 1/5 of women with diabetes. According to other authors, this ailment occurred among women with diabetes a little less frequently [14] or significantly more frequently [13,32] than in our own research. Dyspareunia during sexual intercourse occurring in women with diabetes may be caused by lowered lubrication within the genital organs [30] as well as by psychological factors [32]. The confirmation of this in our research may be the convergent incidence of the occurrence of dyspareunia, lowered lubrication, and reduced sexual satisfaction. A similar convergent incidence of disorders within the scope of these three domains was observed by Nowosielski et al. [14] and Doruk et al. [13], noting that the latter authors observed these disorders in over half of the studied women with diabetes. The differentiation of the obtained study results may have been associated with the selection of the population for the studies.

In the population of men with type 1 diabetes studied by us, the IIEF was used; it demonstrated that the frequency of erection disorders affected half of the studied patients, and this proved to be over two times higher than in men without diabetes (50% vs. 22.6%). A similar frequency of erection disorders among men with type 1 diabetes has been presented by other authors (Dembe et al., 55.1% of the studied subjects [34]; Brooks, 50.0% [35]; Schiavi and Segraves, 50.0% [36]), whereas less frequent occurrence of this disorder was observed by Enzlin et al., 22.0% [19] and Penson et al., 34% [37] and on the basis of the meta-analysis performed by Kouidrat et al., 37.5% [38]. We demonstrated that in men with type 2 diabetes aged 53 (mean), the frequency of occurrence of erection disorders was 81% [17].

The multifactorial analysis in the logistic regression model allowed us to present the statistically significant risk factors for sexual disorders in the studied men and women with type 1 diabetes. The occurrence of sexual functioning disorders demonstrated a significant correlation with sex and with the co-occurrence of diabetes complications (diabetic foot, neuropathy, nephropathy, retinopathy). The probability of the occurrence of sexual disorders proved to be four times higher in men than in women and nearly eight times higher in patients with diabetes complications. A significant intensification of sexual disorders was also demonstrated together with the increase of the concentration of glycated hemoglobin in the blood serum; the odds ratio was 1.81. The highest concentration of HbA1c was observed in women with sexual functioning disorders. This observation corresponds to the study results obtained by Salonia et al. [39] who described the occurrence of a significant correlation between the concentration of HbA1c and sexual disorders in women with diabetes in the luteal phase of the menstrual cycle. The risk factors for the occurrence of sexual disorders both in women and in men with type 1 diabetes have been described in a small number of reports. According to Enzlin et al. [19], the significant risk factors for sexual disorders in men included diabetes complications; such associations were not observed among women with diabetes who reported the occurrence of sexual functioning disorders.

In our research, it was demonstrated that the frequency of occurrence of low mood among women with type 1 diabetes was higher when compared to controls (35% vs 7%). In the present paper, it was concluded that the correlation coefficients in the covariance studies between the BDI score and the studied domains of sexual disorders in women were highly statistically significant. It was therefore proven that the higher the degree of depression intensification in ill women, the more intensified the disorders of sexual functioning as determined using FSFI. In the control group, such a dependency was only observed within the scope of sexual satisfaction and dyspareunia. The research carried out by Bonierbale et al. [40] indicates that in female patients with depression, sexual disorders, especially in terms of sexual desire, occurred significantly more frequently than in women without depression. 

In the men with type 1 diabetes studied by us, symptoms of depression determined on the basis of the BDI score were observed in one in five patients. These results are similar to those described by Enzlin et al. [19], who demonstrated that the frequency of mood disorders among men with type 1 diabetes was 15%. Similar to the case of the studied women, in men with diabetes, we demonstrated the occurrence of significant correlations between the BDI score and sexual disorders demonstrated in the IIEF. Such studies have not been found in the available literature. 

In the studies related to the self-acceptance of illness carried out using the AIS, we demonstrated that the vast majority of the patients studied by us declared acceptance of their illness. It turned out that the higher the declared level of the patient’s acceptance of his or her illness, the lower the intensity of occurrence of sexual functioning disorders, which has been demonstrated in correlation studies. It should be underlined that studies related to the occurrence of sexual functioning disorders in patients with diabetes in connection with their self-acceptance of the illness have not been carried out until now. 

In the patients with diabetes studied by us, the BMI value and the waist-to-hip ratio related to it were significantly higher than in the controls. The possible influence of increased body mass on the obtained results has not been confirmed in the present paper due to the fact that the applied logistic regression analysis did not demonstrate connections between the BMI and the values of FSFI and IIEF. 

We are aware of some limitations of this study. This is one of the first studies in Poland related to both depression and the sexuality of women and men with type 1 diabetes which—thanks to the application of multifactorial statistical analysis—allowed for the specifying of risk factors for sexual disorders. Firstly, the cross-sectional and not prospective nature of the study certainly does not allow for considering other factors which in the context of time could modify sexuality. Secondly, the study sample is too small to generalize the obtained results. Thirdly, as in other self-report inventories (FSFI/IIEF, BDI, and AIS), the scoring systems are subjective in nature. Fourthly, in the case of sexual disorders, the authors did not consider the lack of satisfaction as a factor discriminating the occurrence of clinically significant sexual disorders, although it seems that introducing such a scale would not affect the study results—the aim of the study was the assessment of the occurrence of disorders of particular sexual functions and not their intensity. Fifthly, due to the inability to precisely determine the applied medications and their dosage, the connections between sexual functions and the applied pharmacological treatment have not been taken into consideration in the studied patients.

## 5. Conclusions

Type 1 diabetes leads to sexual disorders which occur in 35% of women and 50% of men. The sexual disorders in patients with type 1 diabetes demonstrate positive correlation with the occurrence of depression, which occurs more frequently in patients with diabetes than in those without it; the mentioned disorders are less intensified in patients who accept their illness. Sexual disorders in patients with diabetes more frequently occur in men, persons with coexisting diabetes complications, and in those with a concentration of glycated hemoglobin exceeding 6.5%.

## Figures and Tables

**Table 1:** Sociodemographic, clinical, and biochemical characteristics of the studied patients.

Parameter		Female			Male		
		Diabetes Type 1 (Group I) (*n* = 57)	Controls (Group CI) (*n* = 43)	*p*	Diabetes Type 1 (Group II) (*n* = 58)	Controls (Group CII) (*n* = 62)	*p*
Age (years)		31.5 ± 8.9	27.7 ± 6.8	0.0583	28.2 ± 7.1	29.5 ± 6.9	0.2501
BMI (kg/m^2^; range)		23.7 ± 3.8; 19.9–32	21.4 ± 1.3; 19.2–23.8	0.0001	26.0 ± 3.6; 19.4–40.7	23.6 ± 1.3; 19.9–29.3	<0.0001
Waist circumference (cm)		79.0 ± 13.3	65.9 ± 3.5	<0.0001	90.6 ± 19.1	88.6 ± 5.5	0.7546
Waist-to-hip ratio; range		0.83 ± 0.11; 0.61–1.09	0.76 ± 0.05; 07–0.84	0.0038	0.89 ± 0.15; 0.67–1.65	0.85 ± 0.04; 0.73–0.91	0.0006
Duration of diabetes (years; range)		9.96 ± 7.37; 1–30			8.64 ± 7.11; 1–28		
Education	Vocational/Primary	13	10	0.8670	17	14	0.5955
	Pre-university	24	20		18	24	
	Higher vocational/University	20	13		23	24	
Place of residence	Urban area	37	30	0.6092	40	39	0.4841
	Rural area	20	13		18	23	
Marital status	Separated/Divorced	18	14	not calculated	21	16	0.4886 **
	Married/In a relationship	39	28		35	43	
	Widow/Widower	0	1		2	3	
Currently working/Not working		43/14	38/5	0.1692 *	48/10	55/7	0.5014 *
Smoking: Never/Past/Present		27/15/15	25/9/9	0.7453 *	15/19/24	32/14/16	0.0151
Alcohol: Drinking/Not drinking		28/29	26/17	0.2599	39/19	38/24	0.4969
Systolic blood pressure (mm Hg)		126.8 ± 13.4	120.3 ± 2.4	0.0054	123.4 ± 9.1	120.5 ± 2.6	0.0368
Diastolic blood pressure (mm Hg)		79.3 ± 9.8	64.3 ± 4.6	<0.0001	78.4 ± 10.7	62.4 ± 3.8	<0.0001
Comorbidities	Hypertension	1			0		
	Coronary artery disease	9			4		
	Heart failure	1			0		
Drugs	Oral antidiabetic/antihypertensive	6/9			4/4		
Diabetic complications: diabetic foot/neuropathy/nephropathy/retinopathy		1/5/0/10			0/2/0/9		
Glucose fasting (mg/dL)		121.4 ± 23.1	84.7 ± 3.7	<0.0001	120.4 ± 25.6	86.0 ± 4.3	<0.0001
HbA1c (%; range)		6.11 ± 0.98; 5.3–7.5			5.96 ± 1.0; 5.1–8.4		
Total cholesterol (mg/dL; range)		157.2 ± 26.4; 100–240	99.0 ± 7.5; 90–120	<0.0001	159.3 ± 27.5 83–226	98.6 ± 9.0; 83–130	<0.0001
LDL-cholesterol (mg/dL; range)		94.1 ± 28.9; 30–156	53.3 ± 6.0; 45–67	<0.0001	96.5 ± 29.9; 40–160	51.2 ± 3.4; 40–61	<0.0001
HDL-cholesterol (mg/dL; range)		60.3 ± 15.5; 35–130	64.0 ± 4.6; 57–74	0.0034	56.8 ± 13.7; 38–90	58.1 ± 4.6; 50–71	0.1866
Triglycerides (mg/dL; range)		132.8 ± 22.9; 50–167	90.8 ± 5.7; 78–102	<0.0001	138.2 ± 25.2; 75–198	86.9 ± 5.7; 77–110	<0.0001

BMI = body mass index, HbA1c = glycated hemoglobin, LDL = low-density lipoprotein, HDL = high-density lipoprotein, *p* = statistical significance of differences, * Yates-corrected Chi-square test, ** Fisher’s exact test; the remaining *p*-values were calculated using the Mann–Whitney *U* test.

**Table 2:** Sexual function and Beck Depression Inventory (BDI) characteristics in diabetic women and controls.

Domains of Sexual Function	Female (Mean ± SD)				Statistical Significance of Difference (*p*)
	Type 1 Diabetics (Group I) (*n* = 57)		Controls (Group CI) (*n* = 43)		
	Mean ± SD	Sexual Dysfunction *n* (%)	Mean ± SD	Sexual Dysfunction *n* (%)	
Desire	4.49 ± 1.28	18 (31.6)	5.54 ± 0.61	0 (0)	0.0001
Arousal	4.86 ± 1.05	11 (19.3)	5.49 ± 0.57	0 (0)	0.0024
Lubrication	4.99 ± 1.00	11 (19.3)	5.56 ± 0.52	0 (0)	0.0073
Orgasm	4.90 ± 1.03	9 (15.8)	5.61 ± 0.51	0 (0)	0.0003
Sexual satisfaction	4.86 ± 1.09	11 (19.3)	5.56 ± 0.55	0 (0)	0.0019
Dyspareunia	4.81 ± 1.08	11 (19.3)	5.56 ± 0.62	2 (4.7)	0.0006
Total FSFI	28.91 ± 5.81	20 (35.1)	33.33 ± 2.96	2 (4.7)	0.0001
BDI *	8.16 ± 8.07		2.79 ± 4.39		0.0001

FSFI = Female Sexual Function Index; * Depression: diabetic patients, *n* = 20 (35.1%); controls, *n* = 3 (7.0%); the *p*-values were calculated using the Mann–Whitney *U* test.

**Table 3:** Sexual function and Beck Depression Inventory (BDI) characteristics in diabetic men and controls.

Domains of Sexual Function	Male (Mean ± SD)		Statistical Significance of Difference (*p*)
	Type 1 Diabetics (Group II) (*n* = 58)	Controls (Group CII) (*n* = 62)	
Erection	24.93 ± 4.94 *	27.82 ± 2.45 *	0.0002
Orgasm	8.62 ± 1.65	9.16 ± 0.96	0.1883
Desire	8.45 ± 1.52	9.15 ± 0.97	0.0172
Sexual satisfaction	12.14 ± 2.41	13.55 ± 1.35	0.0009
Overall satisfaction	8.22 ± 1.81	9.15 ± 1.11	0.0044
BDI	5.95 ± 5.98 **	3.13 ± 3.76 **	<0.001

* Erectile dysfunction: diabetic patients, *n* = 29 (50%), including mild dysfunction in 22 (37.9%) and moderate in 7 (12.1%) patients; controls, *n* = 14 (22.6%) (*p* = 0.0017); ** Depression: diabetic patients, *n* = 13 (22.4%); controls, *n* = 3 (4.8%) (*p* = 0.01).

**Table 4:** Values of the Female Sexual Function Index (FSFI) and the International Index of Erectile Function (IIEF) in diabetic patients with and without complications.

Parameter	Diabetic Female (*n* = 57)		Diabetic Male (*n* = 58)	
	With Complications * (*n* = 16)	Without Complications (*n* = 41)	With Complications * (*n* = 11)	Without Complications (*n* = 47)
FSFI (Total)	23.05 ± 5.58 **	31.19 ± 4.07	-	-
IIEF (Erection)	-	-	18.18 ± 6.72 ***	26.36 ± 3.07

* diabetic foot, neuropathy, nephropathy, retinopathy; ** *p* < 0.0001; *** *p* < 0.001.

**Table 5:** Glycated hemoglobin (HbA1c) distribution in diabetic patients with and without sexual dysfunction.

Parameter	Diabetic Female (*n* = 57)		Diabetic Male (*n* = 58)	
	With Sexual Dysfunction (*n* = 20)	Without Sexual Dysfunction (*n* = 37)	With Sexual Dysfunction (*n* = 29)	Without Sexual Dysfunction (*n* = 29)
HbA1c (%)	6.77 ± 0.84 *	5.76 ± 0.86	6.22 ± 1.27	5.71 ± 0.56

* *p* < 0.0001.

**Table 6:** The relationship between the Female Sexual Function Index (FSFI) or the International Index of Erectile Function (IIEF) and the Beck Depression Inventory and the Acceptance of Illness Scale.

Domains of Sexual Function		Beck Depression Inventory				Acceptance of Illness Scale	
		Diabetic Females (*n* = 57)		Control Females (*n* = 43)		Diabetic Females (*n* = 57)	
		*r*	*p*	*r*	*p*	*r*	*p*
FSFI	Desire	−0.55	<0.0001	−0.10	0.5184	0.61	<0.0001
	Arousal	−0.53	<0.0001	−0.15	0.3438	0.52	<0.0001
	Lubrication	−0.59	<0.0001	−0.18	0.2441	0.58	<0.0001
	Orgasm	−0.57	<0.0001	−0.22	0.1521	0.60	<0.0001
	Sexual satisfaction	−0.57	<0.0001	−0.32	0.0389	0.62	<0.0001
	Dyspareunia	−0.66	<0.0001	−0.37	0.0150	0.70	<0.0001
	Total FSFI	−0.65	<0.0001	−0.23	0.1306	0.68	<0.0001
		Diabetic Males (*n* = 58)		Control Males (*n* = 62)		Diabetic Males (*n* = 58)	
IIEF	Erection	−0.66	<0.0001	−0.28	0.0258	0.59	<0.0001
	Orgasm	−0.55	<0.0001	−0.27	0.0306	0.46	0.0003
	Desire	−0.63	<0.0001	−0.49	<0.0001	0.54	<0.0001
	Sexual satisfaction	−0.63	<0.0001	−0.20	0.1159	0.69	<0.0001
	Overall satisfaction	−0.59	<0.0001	−0.27	0.0362	0.58	<0.0001

*r* = correlation coefficient; *p* = statistical significance of difference.

**Table 7:** Odds ratios and the result of Wald’s significance test obtained from the logistic regression of pathological FSFI and IIEF value occurrences in connection with sex, age, BMI, duration of diabetes, HbA1c, complications, and MAP of the respondents.

FSFI/IIEF	Rating	Standard Error	Wald χ^2^	*p*	Odds Ratio (OR)	95% CI for OR	
Absolute term	−8.58	3.25	6.94	0.0084	0.0002	3 × 10^−7^	−0.1192
Sex	1.44	0.56	6.72	0.0096	4.23	1.40	−12.75
Age	0.05	0.04	1.55	0.2135	1.05	0.97	−1.14
BMI	0.53	0.40	1.76	0.1843	1.69	0.77	−3.71
Duration of diabetes	0.04	0.05	0.66	0.4158	1.04	0.94	−1.15
HbA1c	0.59	0.29	4.04	0.0444	1.81	1.01	−3.24
Diabetic complications	2.07	0.67	9.44	0.0021	7.91	2.08	−30.04
MAP	0.003	0.03	0.01	0.9131	1.00	0.95	−1.05

*p* = statistical significance of difference, BMI = body mass index, HbA1c = glycated hemoglobin, MAP = mean arterial pressure, 95% CI = confidence interval.
